# Correction to ‘Information sensitivity functions to assess parameter information gain and identifiability of dynamical systems’

**DOI:** 10.1098/rsif.2018.0353

**Published:** 2018-06-06

**Authors:** Sanjay Pant

*J. R. Soc. Interface* **15**, 20170871 (Published online 16 May 2018) (doi:10.1098/rsif.2017.0871)

The funding statement and reference [10] should be revised as follows:
